# Dual isoform sequencing reveals complex transcriptomic and epitranscriptomic landscapes of a prototype baculovirus

**DOI:** 10.1038/s41598-022-05457-8

**Published:** 2022-01-25

**Authors:** Gábor Torma, Dóra Tombácz, Norbert Moldován, Ádám Fülöp, István Prazsák, Zsolt Csabai, Michael Snyder, Zsolt Boldogkői

**Affiliations:** 1 Department of Medical Biology, Albert Szent-Györgyi Medical School, University of Szeged, Szeged, 6720 Hungary; 2 Department of Genetics, School of Medicine, Stanford University, Stanford, CA 94305 USA

**Keywords:** Gene expression analysis, Virology, Transcriptomics

## Abstract

In this study, two long-read sequencing (LRS) techniques, MinION from Oxford Nanopore Technologies and Sequel from the Pacific Biosciences, were used for the transcriptional characterization of a prototype baculovirus, *Autographa californica multiple nucleopolyhedrovirus.* LRS is able to read full-length RNA molecules, and thereby distinguish between transcript isoforms, mono- and polycistronic RNAs, and overlapping transcripts. Altogether, we detected 875 transcript species, of which 759 were novel and 116 were annotated previously. These RNA molecules include 41 novel putative protein coding transcripts [each containing 5′-truncated in-frame open reading frames (ORFs), 14 monocistronic transcripts, 99 polygenic RNAs, 101 non-coding RNAs, and 504 untranslated region isoforms. This work also identified novel replication origin-associated transcripts, upstream ORFs, cis-regulatory sequences and poly(A) sites. We also detected RNA methylation in 99 viral genes and RNA hyper-editing in the longer 5′-UTR transcript isoform of the canonical ORF 19 transcript.

## Introduction

*Autographa californica multiple nucleopolyhedrovirus* (AcMNPV) is an insect virus and a member of the *Baculoviridae* family^1^. Recently, a recombinant anti-SARS-CoV-2 nanoparticle vaccine based on this virus has been developed^2^. Two distinct forms of the virion are produced during the infection: the occlusion-derived viruses are surrounded by the envelope containing viral proteins, which ensures their survival even in harsh environments such as the insect’s midgut. On the other hand, budded virions have an envelope and some proteins that facilitate their systemic transmission in the near-neutral environment of the insect tissue^3^. The 134 kbp long, double-stranded circular viral DNA encompasses 150 tightly packed open reading frames (ORFs)^4^. AcMNPV genes are expressed in three phases: early (E), late (L), and very late (VL)^5^. Early transcription [0 to 6 h post infection (p.i.)] produces transcriptional activators^6^ and the molecular machinery of DNA replication^7^. E genes are defined as the ones transcribed by the host RNA polymerase II, which recognizes the TATA promoter elements located upstream of the transcriptional start site (TSS) or located to the arthropod initiator element: CAGT^8^; nonetheless, some E genes lack a canonical initiator sequence or any recognizable promoter motif^9^. After a transitory early/late phase, some E genes cease to express, whereas others are transcribed throughout the entire infection cycle, supposedly because of the presence of both early and late promoters and/or initiators^10^. The L phase starts at the onset of the genome replication (6 to 18 h p.i.). L and VL genes are transcribed by the viral RNA polymerase (RNP), which recognizes a consensus late initiator sequence (TAAG) on the DNA, and starts to synthesize the RNAs from the second nucleotide of the motif (see underlined A)^11,12^. In our previous study, we demonstrated that the longer and shorter 5′-untranslated region (UTR) isoforms of a given late transcript can also originate from a late initiation sequence (LIS)^13^. VL gene expression (18 to 72 h p.i.) is characterized by the synthesis of occlusion body proteins: polyhedrin and p10, and transcription factors such as the very late expression factor 1 (VLF-1). VL genes contain an A/T-rich region^14^ and it is referred to as the ‘burst sequence’ downstream of their LIS, being recognized by VLF-1, which facilitates the high expression of these VL gnes^15^.

Capping the viral RNAs is accomplished by both host and viral proteins: LEF-4 exhibits RNA 5′-triphosphatase and guanylyl transferase activities^16^, whereas MTase (encoded by Ac69) methylates guanosine in the Cap structure^17^. Most of the AcMNPV transcripts contain a canonical polyadenylation signal (PAS) upstream their transcriptional end site (TES). PASs are recognized by the host cleavage and polyadenylation apparatus, which nicks the transcripts in their 3′-UTR regions and carries out a non-templated addition of adenines. Viral (RNP) has also been demonstrated^18^ to catalyze the poly(A) tail formation after transcribing uracil-rich regions, which may lead to alternative terminations in the late transcript species^13^.

Early works leading to the discovery of the mRNA Cap structure have detected a low level of internal 5-methylcitosine (5-mC) in the mammalian and insect cells^19,20^. Later studies have also detected the presence of methylated nucleotides in viral RNAs^21,22^. The presence of 5-mC has been shown to be linked to the metabolic stability of tRNAs^23,24^, and it may act as a suppressor of translation when present at position 34 in eukaryotic tRNA^Leu^^25^. In rRNA, 5-mC methylation has been demonstrated to participate in tRNA recognition and peptidyl transfer^26^. In contrast, little is known about the function of methylation in other non-coding RNAs (ncRNAs) and mRNAs. Methylated viral transcripts have been shown to ablate the activity of dendritic cells^27^ in mammals thus reducing immune response to foreign RNAs. Cytosine-5 methylation of vault ncRNA has been shown to determine its processing into small regulatory transcripts^28^.

RNA editing included the C6 deamination of adenine bases by the adenosine deaminase of the host, which acts on the RNA1 (ADAR1) enzyme. The resulting inosine (I) is recognized as guanine (G) by the reverse transcriptase, producing a G mismatch during cDNA sequencing^29^. Hyper-editing is the same phenomenon occurring in several adenines on the transcript. Hyper-editing may play a role in the cell’s innate immunity^30^, while viruses can evade their inactivation and a strong immune response by the presence of hyper-edited sites on their RNA^31^. Hyper-editing has also been shown to be essential for the replication of some viruses^32^.

Clearly, next-generation sequencing (NGS) techniques provide massive amounts of highly accurate data on the structure and expression of genes^33,34^. However, they are inefficient in identifying UTR isoforms, polycistronic RNAs, transcriptional overlaps, and characterizing gene expression because of the short read lengths produced by these platforms^35^.

The Oxford Nanopore Technologies (ONT) and the Pacific BioSciences (PacBio) long-read sequencing (LRS) platforms can overcome these deficiencies by their ability of sequencing full-length RNA molecules^36^, using cDNA^37^ or direct RNA^38^ (only ONT) sequencing. The ONT MinION platform works by measuring the electric current fluctuations caused by the threading of a single-stranded polynucleotide through a nanopore fixed on a synthetic membrane^37,39^. The ONT MinION technology has no theoretical upper limit regarding its read length, but at present, it falls short of its competitors in respect of base calling precision^40^. Its ability of processing full-length RNAs makes it an optimal choice for discovering novel transcripts and transcript isoforms in well-annotated genomes, and as such, its error-prone base calling is not an important issue^41^. The PacBio approach is based on Single-molecule, Real-time (SMRT) technology. The elongation of the DNA sequence is recorded as light impulses emitted when either of the four fluorescently labelled nucleotides is incorporated into the molecule. The single molecules are made circular by the addition of hairpin adaptors, and therefore sequenced in multiple passes in both forward and reverse orientations.

Reverse transcription (RT) and PCR are inevitable cDNA library preparation steps for both NGS and third-generation sequencing technologies. As discussed in our previous works^13,41,42^, both can lead to artifacts through template switching and false priming. This fact should be taken into consideration during the identification of TSSs and TESs. Direct sequencing of ribonucleotides with the ONT approach allows for the detection of modified and edited bases through the comparison of altered and canonical signals^38,43^, but this approach implies a known location of the modification or editing. It can be circumvented by generating models of the altered signals, and fitting them to the unaltered signal^44^. At the time of writing, only a single model for the detection of 5-methylcitosines has been available for public use^44^.

The structure of AcMNPV transcriptome has already been characterized in a study applying Illumina short-read sequencing (SRS) of the 5′ and 3′ transcript ends^12^ as well as in our work^13^ using third-generation long-read cDNA and direct RNA sequencings. Other studies using microarray^45^, real-time PCR analysis^46^, and Illumina SRS^12^ have focused on the characterization of transcriptional dynamics. The techniques used in these gene expression studies are not well-suited to tackle the structural complexity of the baculovirus transcriptome. Therefore, the aims of this work were to update the AcMNPV transcriptome using a dual LRS approach and to detect RNA methylation and editing by using ONT sequencing.

## Results

### Analysis of AcMNPV transcriptome using third-generation sequencing

**Figure 1:**
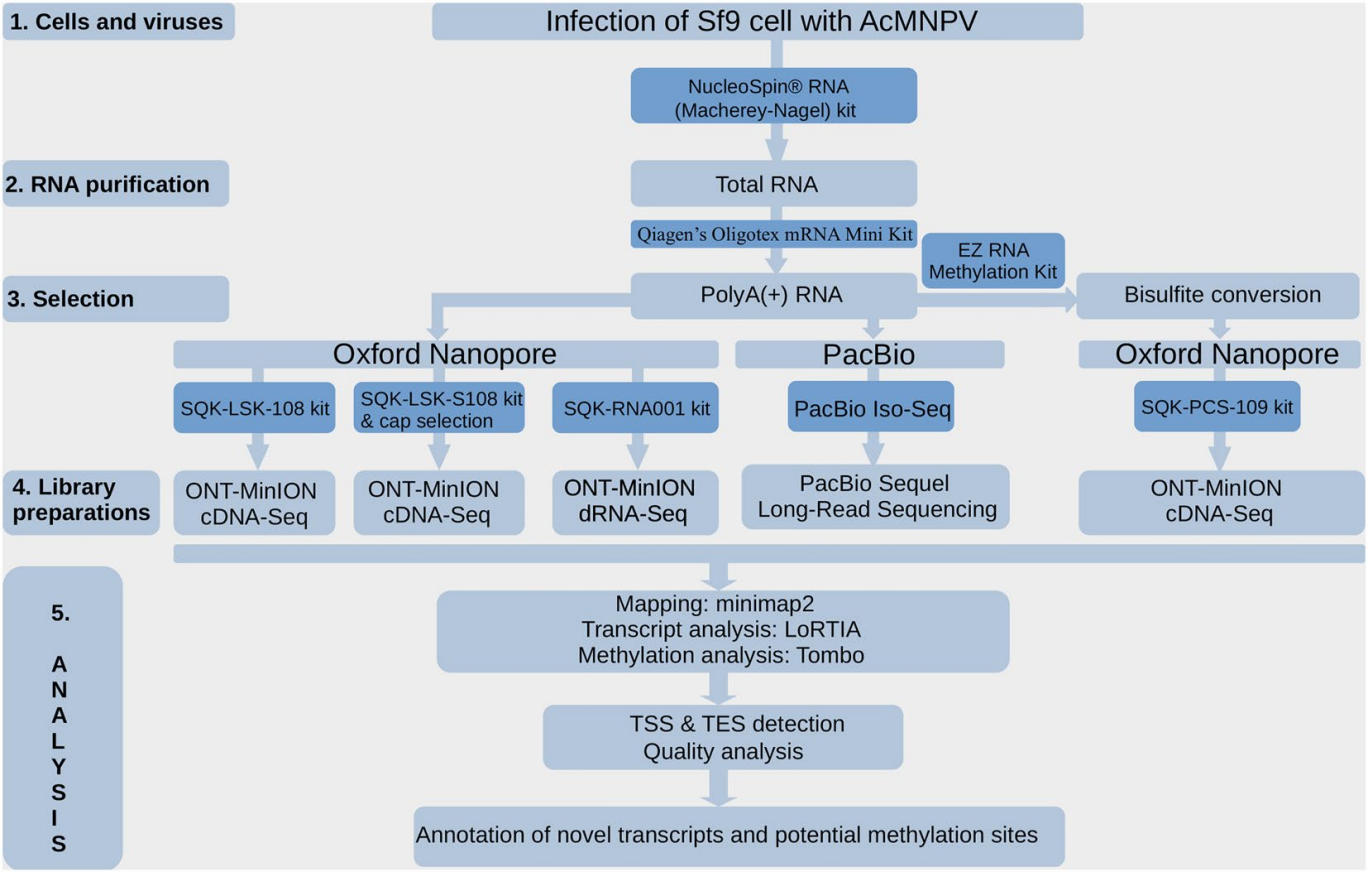
Workflow of transcriptome and epitranscriptome analyses. The types of kits used for each workflow are shown in the dark blue boxes. Spodoptera frugiperda cells were infected with Autographa californica nuclear polyhedrosis virus (AcMNPV). RNA was isolated from the infected cells, and after poly (A) selection, the samples were sequenced using the Oxford Nanopore technologies (ONT) and the Pacific Biosciences (PacBio) Sequel platforms. In the case of ONT, we used PCR-amplified cDNA-seq (SQK-LSK-108, SQK-PCS-109 kit) and dRNA-seq (SQK-RNA001 kit). ONT dRNA-seq was also applied to determine possible methylation sites using the Tombo software. To confirm the methylation sites, bisulfite conversion was also performed using ONT sequencing. The base-called sequences were aligned with the reference genome using the minimap2 long-read mapping software, followed by the determination of TSS and TES positions of each transcript using the LoRTIA program. The illustration was created with Microsoft PowerPoint 2021 software^47^.

In this study, PacBio Sequel and ONT MinION LRS platforms were used to characterize the structure of AcMNPV transcriptome and epitranscriptome (Fig. 1). Sequel sequencing yielded a total of 47,880 Circular Consensus Sequences (CCS), 25,371 of which mapped to the viral genome and 23,884 to the insect host (Sf9 cells) genome. The total read count was less than the sum of the two mapped read counts because of the eliminated chimeric reads formed during library preparation mapped to both of the genomes. The Cap-selected samples yielded a total of 1,830,476 reads, 198,516 of which mapped to the AcMNPV genome and 1,631,960 to the host genome, whereas the non-Cap selected samples yielded 1,119,716 reads, 290,039 mapping to the viral and 760,533 to the host genome. Sequel sequencing yielded longer mean mapped read lengths than the ONT, while the Cap-selected and non-Cap selected ONT reads had similar mapped read lengths (Table 1). The difference in mean read-length between the two platforms is explained by a step during Sequel library preparation used to mitigate the loading bias of PacBio sequencers, resulting in the loss of short cDNAs. Further details on read counts and read lengths are shown in Supplementary Table 1, and the boxplot of Supplementary Fig. 1 presents the distribution of read lengths per sample.

**Table 1:** Read counts and read lengths of the samples used in this study.

Sample	Total read counts	Mapped read counts		AcMNPV mapped read length (nt)		Sf9 mapped read length (nt)	
		AcMNPV	Sf9	Mean	SD	Mean	SD
PacBio	47,880	25,371	23,884	1514.149	742.323	1548.676	777.428
ONT Cap	6,862,026	198,516	1,631,960	554.829	276.372	380.427	293.708
ONT non-Cap	1,119,716	290,039	760,533	562.771	415.693	551.784	462.438
ONT bisulfite conversion	7,077,229	125,448	6,951,781	268.036	134.894	257.178	126.889
ONT dRNA	66,003	2710	63,293	614.559	380.006	595.645	364.374

The LoRTIA software suit (developed in our laboratory) was used to annotate viral transcripts and also our in-house script augmented by manual checking. The criterion of acceptance of TSSs and TESs as true transcript ends was to identify them by the LoRTIA software suite in two amplified ONT samples and in another technique that was either sequel or Cap-sequencing. A more stringent criterion was applied for the non-coding transcripts and 5′-truncated transcripts: in addition to 2 amplified ONT samples, Cap-selection had to be confirmed.

**Figure 2:**
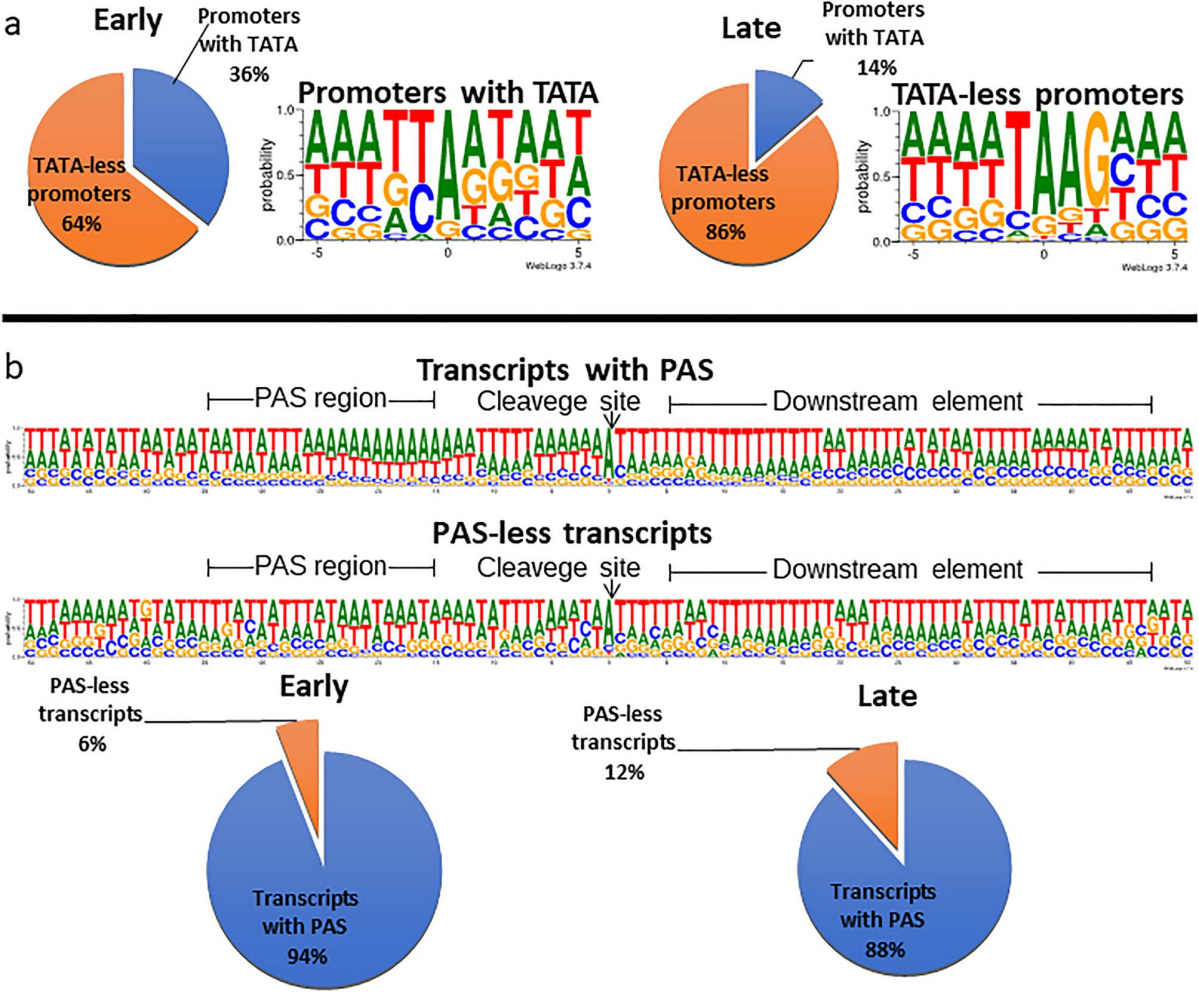
Utilization of TATA box and polyadenylation signal (PAS) in early and late viral transcripts. (**a**) The pie charts show the percentage of TATA (blue) and non-TATA (orange) promoters for early (5 m, 1 h, 2 h, 4 h, and 6 h) and late (16 h, 24 h, 48 h, and 72 h) time points. The pie chart was created with Microsoft Excel 2021 software^48^. (**b**) The weblogo shows the probability of the occurrence of TSSs and nucleotides in their genomic environment. The weblogo shows the poly(A) signal in the downstream element and the probability of occurrence of nucleotides in their vicinity. Pie charts for early (5 m, 1 h, 2 h, 4 h, 6 h) and late (16 h, 24 h, 48 h, 72 h) time points show the percentages of transcripts with poly(A) signal (blue) and without Poly(A) signal (orange). The weblogo image is generated using weblogo 3.0^49^. The pie chart was created with Microsoft Excel 2021 software^48^.

**Figure 3:**
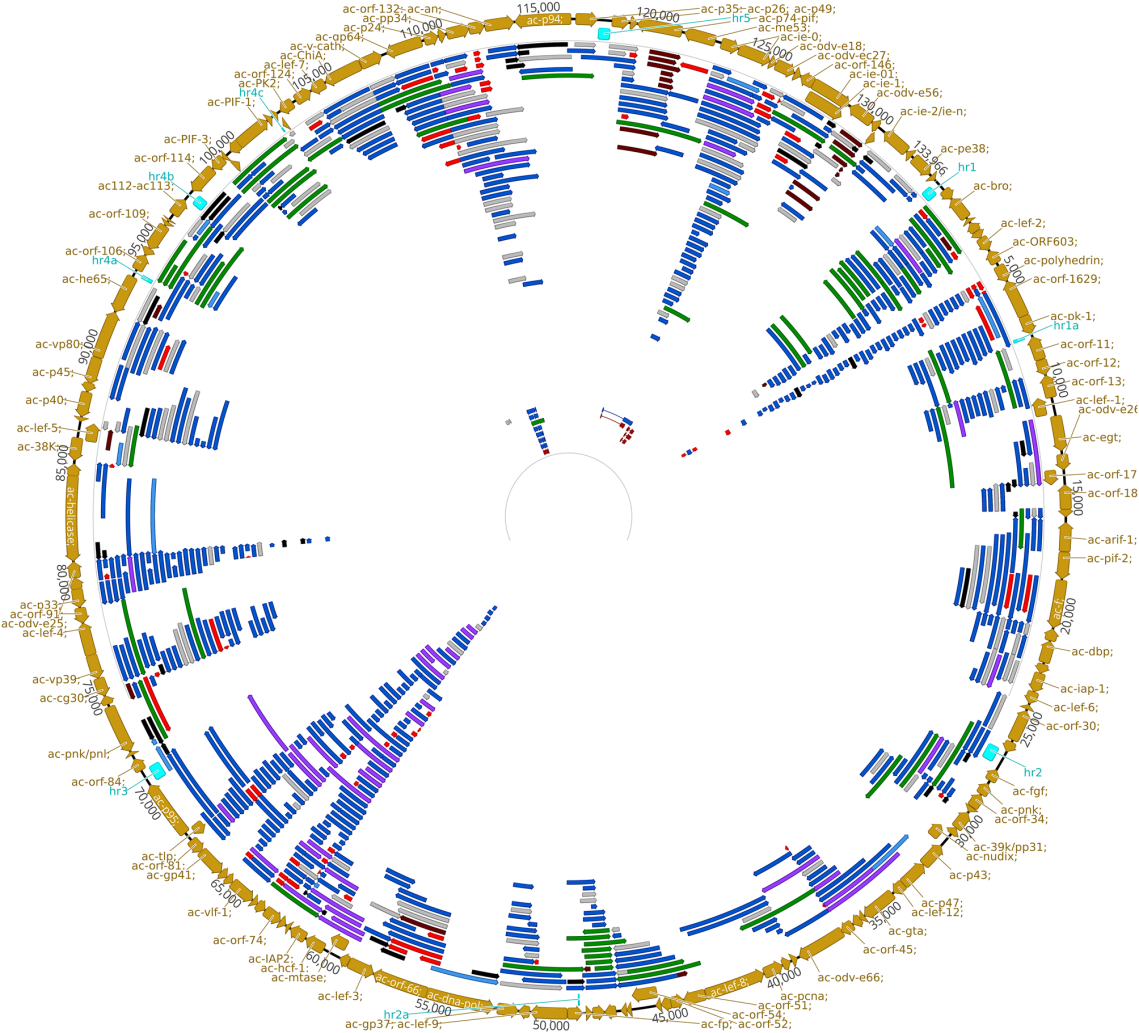
AcMNPV transcripts and isoforms transcribed along the circular genome. Color code. brown arrows: ORFs; aqua rectangles: replication origins; grey: formerly annotated transcripts; light blue: novel monocistronic transcripts; purple: novel polygenic transcripts; green: complex transcripts; red: non-coding transcripts; black: 5′-truncated transcripts; dark blue: TSS and TES isoforms. The figure was created with the Geneious 2021.2.2 software (https://www.geneious.com)^50^.

After the screening, a total of 311 TSSs and 261 TESs (Supplementary Table 2A,B) as well as 13 splice junctions were obtained (Supplementary Table 2C). TATA boxes were identified for 60 TSSs. The mean distance of a TATA box from the TSS was 32 nts. Twenty-two GC boxes were identified, and their average distance from the TSSs was 66 nts. The average distance of the identified 15 CAAT boxes from the TSSs was 108 nucleotides (nts). The canonical CAGT initiators were present in only 6% of TSSs, the TAAG initiators in 61%, and the non-TAAG initiators in 33% of the cases (Fig. 2a). A total of 875 transcript species were detected (Table 2). The full transcript list is available in Supplementary Table 3A, the abundance of transcripts is available in Supplementary Fig. 2 and Supplementary Table 3B, and the transcripts themselves are depicted in Fig. 3.

**Table 2:** The number of previously annotated and novel transcripts of AcMNPV.

Transcript types	Number
Previously annotated transcripts	116
Novel monocistronic transcripts	14
5′-UTR isoforms	164
3′-UTR isoforms	174
5′-UTR isoforms with alternative termination	166
Polygenic transcripts	45
Complex transcripts	54
Non-coding transcripts	78
… of which antisense transcripts	23
Novel putative protein-coding	41

Approximately 80% of TESs were found to contain a canonical PAS at an average distance of 27.23 nts upstream from the TES. In line with previous findings describing arthropod polyadenylation signals^51^, the ± 50 nts surrounding of the viral TESs was characterized by A/U-rich sequences with an increased adenine content immediately upstream of the cleavage site. Intriguingly, sequences harboring a PAS showed a slight increase of adenines between − 26 and − 12 nts upstream from the TES, whereas in the ones without a PAS, this phenomenon could not be observed (Fig. 2b).

### UTR isoforms

In this part of the study, 330 transcript isoforms were found to have longer or shorter 5′-UTRs than the previously annotated transcript encoded by the same genes. Less than half (32.06%) of them showed an E/L initiation region shift when compared to the initiator element (Inr) of their previously annotated transcript. 7.06% of TSS transcript isoforms were revealed to be controlled by TAAG-Inr in the previously annotated isoforms being controlled by non-TAAG-Inr and encoded by the same gene. However, we identified non-TAAG-Inr isoforms in 25% of the transcripts that were previously annotated as TAAG-Inr. This phenomenon suggests that several AcMNPV genes are transcribed by both the host and the viral RNP, resulting in altered 5′-UTR lengths. In addition to the genes described in a previous work^12^, we detected 57 genes, which were transcribed by both the host and the viral RNP (Supplementary Table 4A). Polymorphism in the 5′-UTR length probably has any biological relevance, but we could not exclude that it represents mere transcriptional noise. In many cases, longer 5′-UTRs harbor upstream ORFs (uORFs), which have been shown to alter the translation of the protein coding sequence by ribosome reinitiation, ribosome stalling or disassociation and ribosome bypass^51,52^. We identified 75 gene products containing at least one uORF (Supplementary Table 4B).

All in all, 340 novel TES isoforms were identified in this work, 76.35% of which contained a canonical PAS upstream of their 5′-ends. The phenomenon of nontemplated adenine-addition by the viral RNP has previously been described^18^, and this in-vitro study has also suggested the presence of a T-rich termination signal for this enzyme, and nontemplated thymine addition preceding adenine incorporation. In concordance with this work, we found that 51.85% of the 3′-UTR isoforms with a LIS terminated in the near vicinity (± 3 nts from the TES) of a T-rich region. This finding is in contrast with the 22.51% of the 3′-UTR isoforms with non-TAAG-Inr. However, we could not confirm the presence of nontemplated thymines upstream of the poly(A) tail.

The mean read length of transcripts was 1423.7 nts (σ = 913.190) (Supplementary Table 1). Intriguingly, RNAs transcribed by the viral RNP were on an average of 500 nts longer than those transcribed by the host RNP. The mean 5′-UTR length was 153.06 nts (σ = 270.438), and the mean 3′-UTR length was 529.09 nts (σ = 729.266) both measured from the first ORF overlapped by the transcript. The difference was significant for the transcript length and 3′-UTR length, suggesting the tendency of viral RNP to produce longer RNA molecules.

### Monocistronic transcripts

Several AcMNPV genes lack a precise transcript annotation because of the challenges facing SRS when assembling a genomic region with a complex transcriptional overlapping pattern. Using LRS, we annotated 14 novel monocistronic transcript species with single-nucleotide precision (Supplementary Table 3A). Canonical TATA boxes were observed upstream of the TSSs of ORF85 and ORF112-113. These transcripts started from a non-TAAG-Inr and harbored a canonical PAS upstream of their TES. The transcripts coding for DNA polymerase were initiated at a canonical arthropod initiator (GCATA), while helicase was initiated at a similar but non-canonical sequence (GCAATA). Both DNAPOL and HEL harbored a canonical PAS upstream of their TESs. Nine of the transcripts (ORF1629, P47, ORF72, ORF84, 38K, ORF108, PP34, P49 and ORF154) originated at a TAAG-Inr, PP34 with a canonical CAAT sequence (CCAATC) 87 nts upstream from its TSS, and five of these transcripts had PASs.

### Non-coding transcripts

We detected 101 novel transcript isoform species that did not contain any previously annotated ORFs, two-thirds being longer than 200 nts representing long non-coding RNAs (lncRNAs), while one-third of them fell in the size range of short non-coding RNAs (sncRNAs). We identified 41 sense ncRNAs all of which overlapped a canonical transcript but lacked the stop codons or both the stop codons and a certain part of the 5′-UTRs. Only 10.3% of the ncRNAs had a canonical TATA promoter upstream of their TSSs, whereas 70.5% of them started at a TAAG initiator, which may suggest their late transcription. Twenty-three antisense RNAs (asRNAs) were identified being controlled by their own promoters. These asRNAs were encoded by the complementary DNA strands of 11 genes (Supplementary Table 3A). ORF-60-AS-1 was the only asRNA the promoter of which contained TATA box, whereas 86% of them contained TAAG initiator sequence. All of the ncRNAs were also present in the Cap-selected samples.

### Putative 5′-truncated mRNAs with in-frame ORFs

We detected 41 putative novel genes, which produced 5′-truncated version of the canonical mRNAs and contained shorter in-frame ORFs. Nineteen of these transcripts were initiate at TAAG sequence, 9 of which had a previously annotated isoform (EGT, DNAPOL, HCF1, PNK/PNL, HEL, HE65, 94K, IE1 and IE2) initiated at a non-TAAG-Inr, which may suggest that early genes were partially transcribed by the viral RNP at late time-points. Intriguingly, eleven of the previously annotated transcripts (AC-BRO, POLH, ORF19, PP31, ORF66, ORF84, ODV-E25, BV/ODV-C42, ORF117, CHIT, ODV-EC27) originating at a TAAG sequence had 5′-truncated isoforms that were initiated at non-TAAG-Inrs. It implies the transcription of 5′-truncated isoforms of some late genes by the cellular RNP. These transcripts were all present in the Cap-selected samples.

### Multigenic transcripts

Several long (more than 760 nts) multigenic RNA molecules were detected in the viral transcriptome. We designated polycistronic transcript species to the ones that exclusively contained tandem ORFs, whereas complex transcripts were defined as multigenic transcripts that contained at least one ORF in the opposite orientation as the rest of the ORFs. In this study, we detected 241 polycistronic transcript species containing at least two ORFs. The main initiator motif of these long transcripts was LIS, as 81.74% of the transcripts started at a TAAG sequence. A total of 79 complex transcript species were detected, 21 of which were transcript isoforms, while the rest of them were mapped to unique genomic locations. The longest complex transcript P10-74-ME53-C-1 had only a single sense and two antiparallel ORFs, while ORF51-52-53-LEF-10-ORF54-55-56-C-1 had the highest number of ORFs (6 sense and 1 antiparallel ORFs).

### Replication origin-associated transcripts

The homologous repeat (hr) regions are located in multiple genomic positions in AcMNPV. They are believed to contain the replication origins (Oris). Our LRS approach detected overlapping transcriptional activity at all of the 9 h sequences. However, in the case of h5, LoRTIA did not identify transcripts. Despite this fact, we could detect reads without exact TSSs and TESs. Altogether, 55 transcript species were detected at the hr regions, 50 of which contained a TAAG initiator sequence. Fifteen of these RNAs were polygenic, 32 were TSS variants and 3 were TES isoforms, while 8 were monocistronic transcripts. Most of the overlapping transcripts (12) were transcribed at the genomic junction (at hr1) of the circular viral DNA, 7 of which were complex transcripts, 4 were TSS isoforms, and 1 was a monocistronic RNA.

### Splice isoforms and transcripts with retained introns

Chen et al.^12^ have previously reported twelve introns with an abundance above 1%. We detected five additional introns (Table 3). Twelve of the introns detected in this study contained the canonical GT/AG splice junctions, while a single one a less common GC/AG. Chen and colleagues have associated a spliced antisense transcript to ORF115. We detected 2 similarly positioned RNAs (ORF117-L-SP-1 and ORF117-L-SP-2), and ORF117-L-SP-1 had matching introns with the previously annotated transcript. ORF117-L-SP-2 had the same acceptor site position, but its donor site was located 85 nts downstream from the previously annotated genomic location. We could not precisely annotate the TSS of these transcripts; however, according to our data, it was located upstream of ORF117-L-1’s TSS. Splicing of the ORF117-L-SP-2 led to frame shifting within the previously annotated ORF, and to the generation of a novel 246 nt-long ORF, originated upstream of the original ATG.

**Table 3:** TSS, TES, splice junction, ATG and stop codon positions of the spliced AcMNPV transcripts.

Transcript	TSS	TES	Splice junctions	DNA strand	ATG positions	Stop positions	ORF size (nt)
ORF124-SP	103,694	104,617	104,206..104,360	+	103,864	104,605	589
GP64-SP-AT-1	109,926	108,154	109,165..108,999	−	109,842	108,252	1426
GP64-SP-AT-2	109,926	108,154	109,165..108,991	−	109,842	108,985	685
GP64-SP-AT-3	109,926	108,154	109,786..109,656	−	109,842	108,252	1462
GP64-SP-AT-4	109,926	108,195	109,165..108,991	−	109,842	108,985	685
GP64-SP-AT-5	109,926	108,195	109,786..109,656, 109,165..108,991	−	109,842	108,985	555
GP64-SP-AT-6	109,926	108,195	109,786..109,656	−	109,842	108,985	729
GP64-NC-3	109,926	108,292	109,165..108,991	−	109,842	–	
GP64-P24-GP16-C-SP-1	110,921	108,154	109,786..109,656	−	109,842	108,252	1462
IE01-SP-L-1	122,828	129,066	123,017..127,222	+	122,904	129,016	1909
IE0-NC-1	122,878	128,048	123,017..127,222	+	122,904	–	
E56-SP-AT-2	130,224	128,813	129,634..129,185	−	130,210	129,180	583
E56-SP-AT-1	130,224	128,991	129,634..129,185	−	130,210	129,180	583
ODV-E56-SP-L-AT-1	130,499	128,991	129,634..129185	−	130,210	129,180	583

### Transcriptional overlaps

Theoretically, transcripts can overlap each other in three different ways: convergently, divergently, and parallelly. The AcMNPV genome contains 37 convergent gene pairs. Our LRS approach demonstrated that all of the convergent gene pairs produced transcriptional readthroughs, only 3 pairs of which overlapped exclusively at their 3′-UTRs, while the others overlapped the ORFs (Supplementary Table 5). We detected 32 divergent transcriptional overlap out of the 34 gene pairs, and 84 parallel overlaps out of the 87 gene pairs. We assume that a higher data coverage would detect overlaps in every transcript.

### 5-mC methylation

We used dRNA-Seq and bisulfite conversion data for the detection of methylated nucleotides of AcMNPV transcripts.

#### Tombo analysis

**Figure 4:**
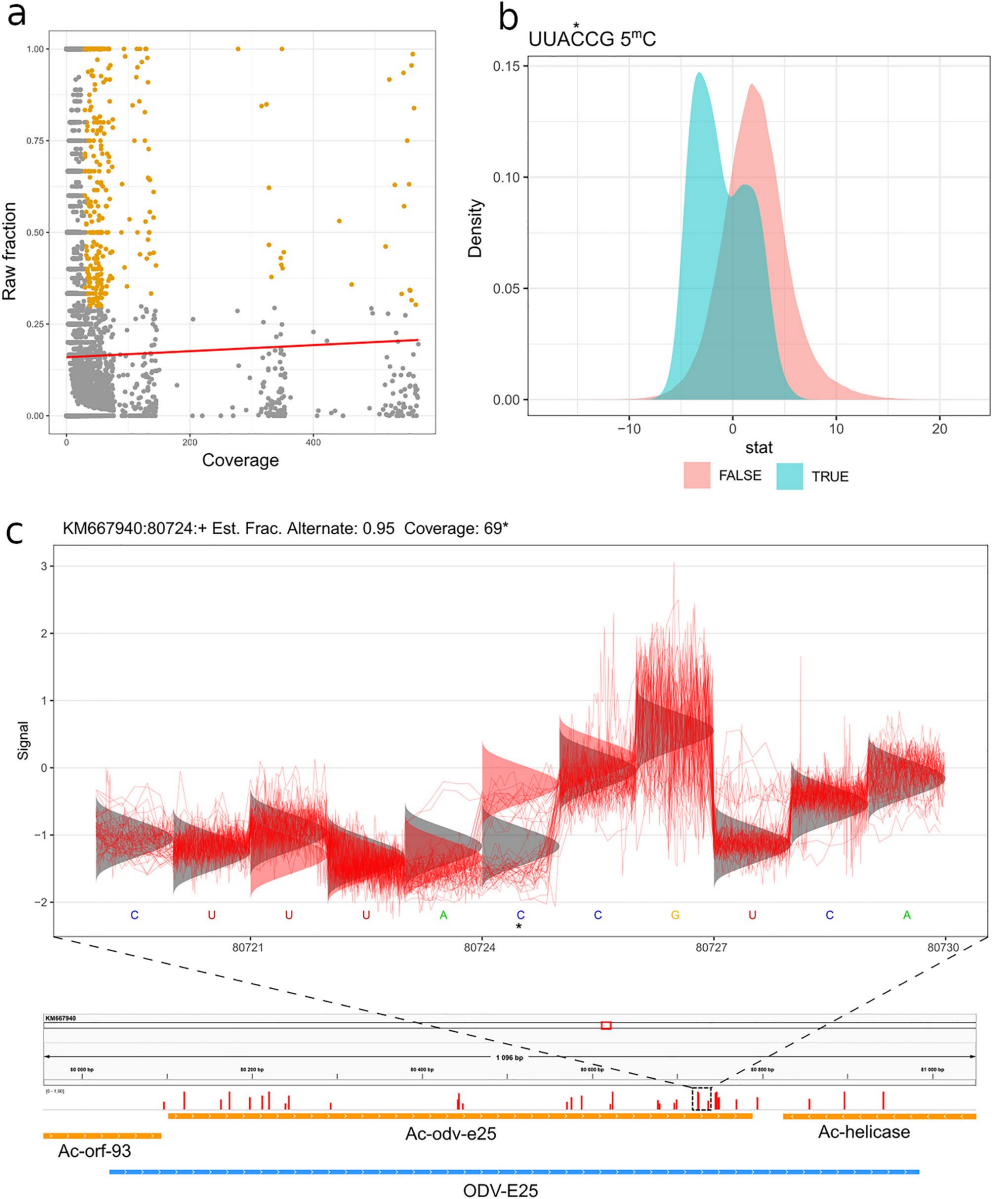
5-mC methylation of AcMNPV transcripts. (**a**) No significant correlation was observed between the coverage and the number of methylated nucleotides in the raw fraction. Yellow dots indicate positions designated for further analysis. The plot was made with the ggplot2 r package^53^. (**b**) Test statistics of UUAC*CG sequence (methylated positions are labelled by asterisks). This panel plots a distribution of test statistics for motif-matching and non-motif-matching sites for all of the provided motives. The plot was made with the ggplot2 r package^53^. (**c**) A putative methylated cytosine of the readthrough part of ODV-E25 transcript (5-mC labelled by asterisk). The red curves indicate the electric signals, while the densities indicate the alternative signal levels. The plot was made with the tombo 1.5.1 software^44^.

Tombo is a software package used for the identification of modified nucleotides from nanopore sequencing data. In order to decrease false positive results in the dRNA-Seq sample, transcripts were filtered with a coverage lower than 30, and the ones the modified fraction of which was less than 30%. We found no significant correlations between the coverage and the number of methylated nucleotides in the raw fraction (Fig. 4a). Using the Tombo software, a possible methylation consensus sequence (UUAC*CG) (the modified C letter is labelled with asterisk) was identified, which indicated the right distribution of log-likelihood ratios (Fig. 4b). Our bisulfite conversion experiment confirmed the methylation of this consensus sequence. The deviation from the canonical C sites could also be clearly detected (Fig. 4c). After identifying the potential false positive sites, we obtained 325 putative 5-mC methylation positions in 12 viral genes (ac-39k, ac-bro, ac-ctl, ac-odv-e25, ac-orf-58, ac-orf-73, ac-orf-74, ac-orf-75, ac-p40, ac-p6.9, ac-polyhedryn, and ac-vp39). The deviation from the canonical C sites could also be clearly detected (Fig. 4c).

#### Bisulfite conversion analysis

Besides the Tombo analysis of dRNA-Seq data, we also carried out bisulfite conversion experiments. While low read counts (2710 viral reads) were obtained in the dRNA-Seq, a much higher read count (125,448 reads) was generated by the bisulfite sequencing. Furthermore, in the latter method, positive control (non-converted samples) was also used. We could confirm 234 methylated positions out of the 325 (identified by Tombo analysis) with the bisulfate sequencing. In order to decrease the false positive results, we set a coverage of 25 as the threshold for the bisulfate analysis. Altogether, 7897 putative methylation positions were identified in the transcripts of 99 genes (Supplementary Table 6 and Supplementary Fig. 3). We detected 31 potential cytosine positions in the 3′-UTR of the ac-Orf-12 transcript, which were always unconverted, and therefore methylated. Altogether, 88% of the examined potential methylation positions (positions the coverage of which was at least 25) were located at the coding regions and 21% at the UTRs.

### A to I RNA hyper-editing

**Figure 5:**
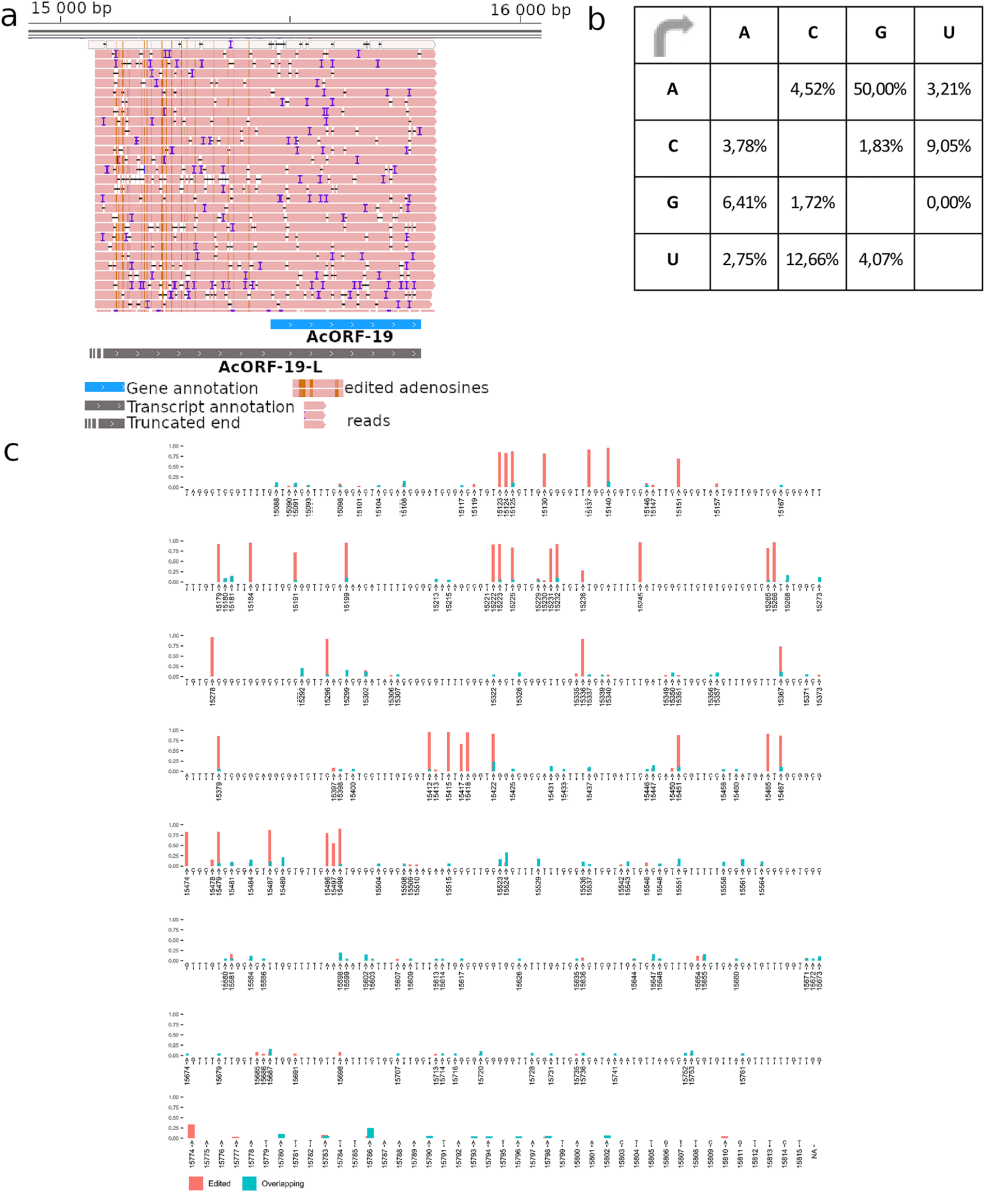
Hyper-editing of AcORF-19 transcript. (**a**) A- > I hyper-edited AcORF-19 reads. The image shows the AcORF19-L transcript reads in pink. The brown lines on the reads indicate base modifications. These modifications are located in the 5′-UTR of the canonical transcript. The reads were visualized with IGV 2.11.3 software^55^. (**b**) Substitution matrix of AcORF-19 reads. Reference nucleotides are found on the left side of the substitution matrix, while the right side of the matrix contains the percentage of nucleotides. It can be seen that that more than 50% of them are G letter. The substitution matrix was created with Microsoft Excel 2021 software^48^. (**c**) The sites and frequency of A- > G substitution on AcORF-19 transcript (indicated on the genomic region encoding this transcript). High-frequency substitutions indicate A- > G editing events, whereas low-frequency substitutions indicate sequencing errors. Red color indicates the high, whereas blue color shows the low frequency of editing. The plot was made with the ggplot2 r package^53^.

Reads of ORF19-L showed a high frequency of A to I (read as A to G by the sequencing) substitution, which was not present in overlapping reads. We found that 50% of all substitutions were A to G (Fig. 5a,b) for ORF19, which was significantly higher than the 16.9% for overlapping transcripts in the same region (p < 0.0001, sided Fisher’s exact test) (Fig. 5c). A substitution threshold of 16.9% was set to distinguish possible edited bases from the noise of sequencing inaccuracy. Our results showed that 18% of all adenines of ORF19-L presented a high level (x̅ = 0.839, σ = 0.153), while 4% of adenines of the overlapping reads presented a low level of A to G editing (x̅ = 0.224, σ = 0.051). To identify the presence of a possible editing motif recognized by ADAR, we calculated the base frequency in the ± 5 nts surrounding the edited A. It has been previously demonstrated that a G-enriched neighborhood and an upstream U stabilize the RNA-ADAR complex in mammalian cells^54^. We detected a significantly higher frequency of Us (*χ*^2^(1, N = 79,455) = 79,338.023, p < 0.01) right upstream of the edited base, while the frequency of Gs was only slightly higher (*χ*^2^(1, N = 79,454) = 79,340.021, p < 0.05) at the + 5 position downstream of the edited base.

## Discussion

The standard next-generation sequencing techniques are limited by short read length because the fragmented sequences have to be re-assembled computationally, during which a significant amount of valuable information on the transcriptome is lost. LRS is particularly useful for the analysis of nested and alternatively spliced transcripts. In this study, we applied two LRS techniques, SMRT Sequel platform from PacBio and MinION platform from ONT for profiling the AcMNPV transcriptome. We carried out amplified and direct RNA sequencing on ONT platform. Altogether, we identified 876 novel transcript species, including mRNAs, ncRNAs, mono- and polygenic transcript species, transcript isoforms, and novel splice sites.

A stepwise truncation on both ends of the transcripts can be observed in several genomic regions. This has been shown to be present in other viruses^56^ as well; however, many studies can confuse this phenomenon with RNA degradation or PCR artefacts, especially if it is present on the 5′-end of the reads. AcMNPV offers a unique support of the existence of these variable UTR isoforms by the presence of a LIS located at the TSS. The same TSSs and TESs are used by multiple transcript isoforms of neighboring, or in some cases, distant genes, resulting in polycistronic and complex transcript isoforms. This organization of the transcriptome, especially the intensive usage of the same TSS for multiple transcript isoforms containing varying TESs, is uncommon in herpesviruses^57^; however, we observed a somewhat similar pattern of TSS/TES usage in African swine fever virus (ASFV)^58^, which is related to insect viruses.

Several multigenic transcripts were detected in AcMNPV. Operons encoding multigenic transcripts represents the basic organization principle of prokaryotic genome, but they are rare in eukaryotes the reason for which is that in bacteria the Shine–Dalgarno sequences allow the translation of every gene in the mRNA^59^, but in eukaryotes only the most upstream gene of a multigenic transcript is translated because of the Cap-dependent initiation system. However, polycistronic RNAs are very common in eukaryotic viruses^60^. The function of these multigenic transcripts are currently unknown because we have no evidence for the translation of downstream genes. It is hypothesized that the transcriptional readthrough in tandem genes (and also on convergent genes) plays a role in a transcription interference-based mechanism^61^.

We observed that many of the longer TSS isoforms contained uORFs in their 5′-UTR, which may play a role in the regulation of translation^52,62^. These transcripts are also involved in the formation transcriptional overlaps. AcMNPV resembles to ASFV and vaccinia virus and differs from herpesviruses in that it exhibits higher heterogeneity in their TESs than TSSs. The alternative use of 3′-UTRs generates long tail-to-tail and tail-to-head transcriptional overlaps. This part of the transcripts may contain cis-regulatory elements, which can bind to regulatory proteins or micro RNAs thereby controlling the translation and the decay of mRNAs^63^.

In this study, we detected novel promoters, Inr sequences and poly(A) sites. Additionally, we identified TAAG-Inr motifs, which bind viral RNP at late and very late phase of viral life cycle, and non-TAAG-Inr motifs recognized by both viral and host RNPs at early time points. Our results clearly demonstrate that viral RNP generates longer transcripts than the host RNP. The 3′-cleavage of the viral RNAs and the formation of a poly(A) tails are carried out by polyadenylation machinery of both the host and the virus, although the latter is not well understood.

AcMNPV contains 9 AT-rich repetitive sequences (hr regions), which are thought to be replication origins^64,65^. However, others have demonstrated that none of them is essential for the viral replication^66^. We detected overlapping transcription from each hr region. They are assumed to play a role in the regulation of replication^67^. Such transcripts have been identified in other viruses as well such as herpesviruses^68^.

We describe several putative 5′-truncated mRNA molecules containing nested in-frame ORFs. This phenomenon has been described in other viruses^58,69^. Further studies are needed to determine whether these transcripts carry the information of N-terminally-truncated polypeptides. If so, this kind of nested transcription significantly increases the coding potential of viruses. In this part of the work, we detected a large number of low-abundance transcript isoforms; nonetheless, their potential functional significance has to be ascertained.

Theoretically, the electric signals (squiggles) generated by the nanopore sequencing of native RNAs might be used to identify 5-mC modifications. Currently, only the Tombo package provides support for this, but our results show that this software tends to produce false positive results. We also obtained false negative results with the Combo software, but it is explained by the low dRNA-Seq data coverage. In order to exclude the false results, we also applied the traditional bisulfate conversion method, which converts non-methylated cytosines with 99.5% efficiency. We could validate 70% of the methylation position obtained by Tombo using bisulfate sequencing. With this approach, we detected 5-mC methylation in transcripts of 99 AcMNPV genes and identified the UUACCG sequence, which is assumed to be a methylation consensus sequence. We found that the majority of methylated positions were located in GC-rich genomic regions. This phenomenon has already been described in mammalian animals^70^. Yang and co-workers have demonstrated that 5-mC bases enhance the nuclear export via the ALYREF adapter protein in mammalian cells^70^. Boyne and colleagues have come to the same conclusion in Kaposi’s sarcoma-associated herpesvirus^71^. ALYREF is also present in Arthropods. We assume that similarly to the mammalian cells^70^ and the Kaposi’s sarcoma virus^71^, the methylation of AcMNPV mRNAs may play a role in nuclear export.

We detected A to I hyper-editing in the 5′-UTR region of the longer TSS isoform of ORF19 canonical transcript. In cellular organism, this process plays an important role in innate immunity^30^, which is unlikely to be the case in AcMNPV. A-I editing is thought to decrease the affinity of antisense transcripts to the complementary mRNAs through inhibiting the binding of dsRNA nucleases (such as RNase)^72^. Since cDNA-Seq-based editing detection is still in its infancy, our results need further confirmation.

## Materials and methods

### Cells and viral infection

AcMNPV expressing *lac*Z gene (*β*gal-AcMNPV) was propagated on the Sf9 cell line (both kindly provided by Ernő Duda Jr., Solvo Biotechnology, Hungary). Cells were cultivated in 200 mL of GIBCO Sf-900 II SFM insect cell medium (Thermo Fisher Scientific) in a Corning spinner flask (Merck) at 70 rpm and 26 °C, and they were infected with a viral titer of 2 multiplicity of infection (MOI = plaque-forming units per cell). A 5 mL sample was measured and centrifuged at 2000 rpm at 4 °C at nine consecutive time points after inoculation (5 min, 1 h, 2 h, 4 h, 6 h, 16 h, 24 h, 48 h, and 72 h), followed by washing with PBS and centrifuged again. Pellets were stored at − 80 °C until use.

### RNA purification

Total RNA was isolated using the Nucleospin RNA Kit (Macherey–Nagel) according to the manufacturer’s instruction. In short, infected cells were collected by centrifugation, and the cell membrane was disrupted by the addition of lysis buffer (provided in the kit). Genomic DNA was digested using the RNase-free rDNase solution (supplied with the kit). Samples were eluted in a total volume of 50 μL nuclease free water. To eliminate residual DNA contamination, samples were treated with the TURBO DNA-free Kit (Thermo Fisher Scientific). The RNA concentration was measured by Qubit 2.0 Fluorometer (Thermo Fisher Scientific) using the Qubit RNA BR Assay Kit (Thermo Fisher Scientific).

### Poly(A) selection

Thirty-five μg of total RNA was pipetted in separate DNA LoBind Eppendorf tubes (Merck) from every time point. The poly(A)^+^ RNA fraction was isolated from the samples using the Oligotex mRNA Mini Kit (Qiagen). The concentrations of the samples were measured by Qubit 2.0 using the Qubit RNA HS Assay Kit (Thermo Fisher Scientific). RNAs were stored at − 80 °C until use.

### Cap-selection, library preparation and sequencing

Cap-selection was carried out with the aim to validate the 5′-ends of the transcripts. For this, we used the TelopPrime Full-Length cDNA Amplification Kit of Lexogen. The starting material was a total RNA mixture (containing samples from 1, 2, 4, 6, 16, 24, 48 and 72 h p.i.). The cDNA generation was carried out according to the recommendations from the manual of Lexogen. Detailed protocol can be found in our earlier published data paper^73^. The cDNA sample was used to produce a sequencing library with the ONT Ligation Sequencing Kit 1D (SQK-LSK108) following the last steps (end-repair and 1D adapter ligation) of ONT’s 1D Strand switching cDNA by ligation method (Version: SSE_9011_v108_revS_18Oct2016). Sequencing was performed on ONT R9.4 SpotON Flow Cells.

### Barcoding

For the kinetic characterization of the viral transcripts, RNA samples from different time points (1, 2, 4, 6, 16, 24, 48 and 72 h p.i.) were used individually for the production of cDNA libraries for the Nanopore sequencing. Libraries were labeled with barcodes applying a combined protocol: the ONT’s 1D Strand switching cDNA by ligation method was used until the end repair step, which was followed by the 1D PCR barcoding (96) genomic DNA protocol (version: PBGE96_9015_v108_revS_18Oct2016, updated 25/10/2017) starting with the Barcode Adapter ligation step^73^. Adapters (Table 4) were ligated to the cDNAs with Blunt/TA Ligase Master Mix (New England Biolabs). PCR reactions were carried out to amplify the adapter-ligated samples with KAPA HiFi DNA polymerase (Kapa Biosystems) and PCR barcodes (ONT). After PCR, samples were mixed and the second end-prep an adapter ligation were carried out according to the ONT’s 1D Strand switching cDNA by ligation method. Sequencing of the mixtures from the barcode labeled samples were performed on ONT R9.4 SpotON Flow Cells.

**Table 4:** Summary table of barcodes used for labeling the samples of different time points after infection.

Sample	Barcode component (ONT PCR Barcoding Kit 96)	Barcode sequence
1 h	BC26	CATACAGCGACTACGCATTCTCAT
2 h	BC27	CGACGGTTAGATTCACCTCTTACA
4 h	BC28	TGAAACCTAAGAAGGCACCGTATC
6 h	BC29	CTAGACACCTTGGGTTGACAGACC
16 h	BC30	TCAGTGAGGATCTACTTCGACCCA
24 h	BC31	TGCGTACAGCAATCAGTTACATTG
48 h	BC32	CCAGTAGAAGTCCGACAACGTCAT
72 h	BC33	CAGACTTGGTACGGTTGGGTAACT

### Bisulfite conversion

RNA bisulfite conversion was carried out for the detection of the methylation frequency (5mC) of AcMNPV transcripts. An RNA mixture containing equal amount of RNA from each examined time point (1, 2, 4, 6, 16, 24, 48 and 72 h p.i.) and the EZ RNA Methylation Kit (Zymo Research) were used for this experiment. Bisulfite conversion was carried out according to the Kit’s manual. In short, 200 ng RNA was mixed with the RNA Conversion Reagent, and then, they were incubated in a PCR cycler (Veriti, Applied Biosystems) at 70 °C for 5 min (denaturation) and then at 54 °C for 45 min (bisulfite conversion). The mixture was cooled down to 4 °C, which was followed by an in-column desulfonation using the Zymo-Spin IC Column. First, the RNA Binding Buffer (part of the Kit), then the RNA sample, and finally, 100% ethanol were loaded to the column, then after a brief mixing, the sample was centrifuged at 13,000×*g* for 30 s. RNA Wash Buffer was added to the column, which was followed by a centrifugation (13,000×*g* for 30 s). Afterwards, desulfonation was carried out with the addition of the Kit’s Desulfonation Buffer to the column. The sample was incubated at room temperature (24 °C) for 30 min, which was followed by centrifugation (13,000×*g* for 30 s). The column was washed twice using the RNA Wash Buffer. Finally, the bisulfite-converted RNA was eluted in 15 μL of DNase/RNase-free water. The RNA was stored at − 80 °C until further usage.

### ONT MinION sequencing

#### Amplified cDNA sequencing

The cDNA library was prepared using the Ligation Sequencing Kit (SQK-LSK108; Oxford Nanopore Technologies) following the modified 1D strand switching cDNA by ligation protocol. Briefly: End repair was carried out on Cap-selected and barcoded samples using NEBNext End repair/dA-tailing Module (New England Biolabs) followed by adapter ligation using adapters (supplied in the kit) and NEB Blunt/TA Ligase Master Mix (New England Biolabs). The cDNA sample was purified between each step using Agencourt AMPure XP magnetic beads (Beckman Coulter), and the library concentration was determined using Qubit 2.0 Fluorometer (through use of the Qubit (ds)DNA HS Assay Kit (Thermo Fisher Scientific). The samples were loaded on R9.4 SpotON Flow Cells.

#### Direct RNA sequencing

The Direct RNA Sequencing (DRS) protocol (Version: DRS_9026_v1_revM_15Dec2016) from ONT was used to generate non-amplified sequencing libraries. For this, we used a mixture from seven total RNA samples (1, 2, 4, 6, 16, 24, 48 and 72 h p.i.). The poly(A)^+^ RNA fraction from this mixture was isolated using the Qiagen’s Oligotex protocol, as we described above. For the DRS library preparation, a 100 ng sample was used. It was mixed with the ONT’s oligo(dT)-adapter (ONT Direct RNA Sequencing Kit; SQK-RNA001) and with T4 DNA ligase New England BioLabs. Incubation was carried out at room temperature for 10 min, and it was followed by the first strand cDNA synthesis using the SuperScript III Reverse Transcriptase enzyme (Life Technologies), according to the DRS protocol^73^: briefly, a 50 min incubation at 50 °C was followed by an inactivation step at 70 °C for 10 min. Sequencing adapters (DRS kit) were ligated to the samples at room temperature for 10 min with the T4 DNA ligase enzyme and NEBNext Quick Ligation Reaction Buffer (New England BioLabs). Libraries were sequenced on an R9.4 SpotON Flow Cell.

The cDNAs and the sequencing ready cDNA libraries were washed using AMPure XP beads (Agencourt, Beckman Coulter) after every enzymatic reaction. The samples for dRNA sequencing were handled with RNase OUT-treated (40 U/μL; Life Technologies) AMPure XP beads. The library concentrations were measured with Qubit 2.0 and Qubit dsDNA HS Assay Kit.

#### cDNA-PCR Sequencing

A cDNA library from the bisulfite-converted sample was generated for MinION sequencing by using the cDNA-PCR Sequencing Kit (SQK-PCS109, Version: PCS_9085_v109_revM_14Aug2019), as follows: the primer (VNP) and dNTP (both from the Kit) were mixed with 50 ng bisulfite-converted RNA, and then, the mixture was incubated at 65 °C for 5 min. This protocol was followed by the addition of RT buffer, RNaseOUT, nuclease-free water, and strand-switching primer to the sample. They were incubated at 40 °C for 2 min, and then, Maxima H Minus Reverse Transcriptase (Thermo Scientific) was measured into the RT mix. Reverse transcription was carried out at 42 °C for 90 min. The RT enzyme was inactivated by heating the sample to 85 °C for 5 min. For the amplification of the first-strand cDNA sample, LongAmp Taq Master Mix (New England Biolabs), cDNA Primer (cPRM, ONT Kit) and Nuclease-free water (Invitrogen) were added. The following settings was used: initial denaturation (95 °C, 30 s), 16 cycles of Denaturation (95 °C, 10 s)—Annealing (62 °C, 15 s)—Extension (65 °C, 4 min), and final extension (65 °C, 6 min). The PCR product was treated with exonuclease (NEB) and was then incubated at 37 °C for 15 min, and 80 °C for 15 min. AMPure XP Beads were used for purification, and the clean samples were eluted. The concentrations of the libraries were detected by Qubit 4.0 and Qubit 1× dsDNA HS Assay Kit, and then, they were loaded to an R9.4 SpotON Flow Cell.

### PacBio sequel sequencing

The cDNAs were produced from the Poly(A) + RNA samples. For this, the SMARTer PCR cDNA Synthesis Kit (Clontech) and the ‘Isoform Sequencing (Iso-Seq) protocol without size selection’ (PacBio) were used. First, the Poly(A) + RNA and the 3′ SMART^®^ CDS [oligo(dT)] Primer II A (SMARTer Kit) were mixed. The mixture was incubated in a thermal cycler at 72 °C for 3 min with slow ramp to 42 °C (0.1 °C/s), then it was hold at 42 °C for 2 min. A mix, containing 5 × First-strand Buffer, DTT, dNTP (10 mM), SMARTer II A Oligonucleotide, SMARTScribe Reverse Transcriptase (both from SMARTer kit) and RNase Inhibitor (Applied Biosystems) was heated to 42 °C and then it was measured into the RNA sample containing tube. Then, this sample was incubated at 42 °C for 90 min, then the enzymatic reaction was terminated at 70 °C for 10 min. The cDNA sample was amplified using KAPA HiFi Enzyme (Kapa Biosystems). The initial denaturation was at 95 °C for 2 min, and then 16 cycles at 98 °C for 20 s, 65 °C for 15 s and 72 °C for 4 min. The final extension was carried out at 72 °C for 5 min.

Five-hundred ng amplified cDNA sample was used to produce PacBio SMRTbell templates for sequencing on the Sequel platform. First, for repairing the DNA damages, the DNA Damage Repair Buffer, NAD+, ATP high, dNTP and DNA Damage Repair Mix 37 °C for 20 min (both from the PacBio Template Prep Kit) were mixed with the cDNA, and they were incubated at 37 °C for 20 min. This step was followed by the end-repair using the End Repair Mix (PacBio Template Prep Kit). Adapters were ligated to the repaired cDNAs with the PacBio’s ligase enzyme and ATP low at 25 °C for 15 min. The incorrect SMRTbell libraries were removed with exonuclease treatment [ExoIII and ExoVII enzymes (from the Template Prep Kit) were added] at 37 °C for 1 h. AMPure^®^ PB (PacBio) bead purification steps were performed after each of the enzymatic steps. The concentration of PacBio SMRTbell template was measured using Qubit fluorometer and Qubit dsDNA HS Assay Kit. SMRTbell libraries were annealed to the sequencing primer v3 and bound to Sequel DNA polymerase 2.0 for sequencing using the Sequel Binding Kit 2.0 (PacBio, 100-862-200), and then, the library-polymerase complex was bound to MagBeads using the PacBio’s MagBead Binding Kit. The amount of the primer for the annealing and the polymerase for the binding were determined by the PacBio IsoSeq Binding Calculator (Sample Setup Module, PacBio SMRT Link), by adding the concentrations and the average insert sizes of SMRTbell templates (MagBead was selected^74^). The total amount of the MagBead-bound complex was loaded onto the Sequel SMRT Cell 1 M v2. One SMRT Cell was run on the Sequel sequencer. The consensus reads (ROIs) were created using SMRT Link5.0.1.9585.

### Data analysis and alignment

Barcoded reads were demultiplexed (ONT’s software) into 9 separate time points and an additional category. Reads with a > 7 quality score of both the Cap-selected and the demultiplexed barcoded datasets were aligned to the circularized genome of AcMNPV strain E2 (GeneBank accession: KM667940.1) and the host cell genome (*Spodoptera frugiperda* isolate Sf9; BioProject accession: PRJNA380964) using Minimap2 v.2.11^75^.

Annotation of the TSSs, TESs, and introns was performed using the LoRTIA software suite (https://github.com/zsolt-balazs/LoRTIA) with specific settings for each sample type (Table 1).

We used SeqTools, our in-house scripts for the generation of the descriptive quality statistics of reads (ReadStatistics) and for the analysis of promoters (MotifFinder), which are available on GitHub: https://github.com/moldovannorbert/seqtools.

In this study, the LoRTIA (https://github.com/zsolt-balazs/LoRTIA, v.0.9.9) pipeline developed in our laboratory was used for the identification of transcripts and transcript isoforms, as was described earlier^73^. Briefly, sequencing adapters and the homopolymer A sequences were checked by the LoRTIA software for the detection of TSS and TES, respectively. For the elimination of false transcript ends, the putative TSSs and TESs were tested against the Poisson distribution (using Bonferroni correction). Introns were identified by applying the following criteria: they have one of the three most frequent splice consensus sequences (GT/AG, GC/AG, AT/AC), and their frequency exceed 1‰ compared to the local coverage (Table 5).

**Table 5:** Settings of the LoRTIA software suite for each sample type.

Sample	5′ adapter	5′ min score	3′ adapter	3′ min score	Search distance	
PacBio Sequel	AGAGTACATGGG	16	AAAAAAAAAAAAAAA	18	15	
ONT Cap	Default	Default	Default	Default	Default	
ONT non-Cap	TGCCATTAGGCCGGG	14	AAAAAAAAAAAAAAA	14	15	

For transcript isoform annotation, TSSs and TESs were selected that were present in at least two samples, while introns were selected if they were present in at least two samples and if their orientation matched the orientation of reads in which they were present, as the LoRTIA software is blind for the orientation of the reads when looking for introns. Transcript isoforms were annotated for each sample using these features and the *Transcript Annotator* module of LoRTIA.

A read was considered a transcript isoform if it started in the ± 5 nt vicinity of a TSS and if it ended in the ± 5 nt vicinity of a TES. Transcripts enclosing the same ORFs as a previously annotated transcript but starting upstream of its TSS were denoted longer (L) 5′-UTR isoforms, while those starting downstream, shorter (S) 5′-UTR isoforms. Transcripts with the same ORFs as a previously annotated transcript but ending upstream or downstream of its TES were denoted transcript isoforms with alternative termination (AT). Transcripts with longer 5′ or 3′-UTRs overlapping multiple ORFs in the same orientation were considered polygenic. If a TSS of a novel transcript isoform was positioned downstream of a previously described ORF’s AUG, with an alternative in-frame start codon downstream from the TSS, the isoform was considered putative protein coding transcript, while those without a 5′-truncated ORF were considered 5′-truncated (TR) non-coding transcripts. Both of these transcript species are conterminal with their previously annotated isoforms. If a transcript isoform started in the same TSS as a previously described protein coding transcript, but its TES was located upstream of the stop codon of previously described ORF, the novel transcript was denoted as non-coding (NC). Transcripts in the opposite orientation of an annotated transcript were named non-coding antisense (AS) transcripts. Very long transcripts overlapping multiple ORFs in different orientation were denoted as complex (C) transcripts. Any other transcript configuration not containing a previously annotated ORF was denoted as NC.

### Detection of RNA modifications

To detect the modifications in the RNA nucleotides, we base-called the raw fast5 files of our previously published direct RNA sequencing dataset deposited in the European Nucleotide Archive under sample accession SAMEA10458962. Modification detection was performed by Tombo software suite^43^ (v.1.3.1.).

### Ethics declaration

Neither human nor animal experiments were applied in this study.

## Supplementary Information

Supplementary Table 1.

Supplementary Table 2.

Supplementary Table 3.

Supplementary Table 4.

Supplementary Table 5.

Supplementary Table 6.

Supplementary Figures.

## Data Availability

The sequencing data and the transcriptome assembly have been uploaded to the European Nucleotide Archive under the project accession number PRJEB25619 for samples at separate time points and PRJEB24943 for the mixed and Cap-selected samples.

## Code availability

**Accession codes**: The LoRTIA software suite is available on GitHub: https://github.com/zsolt-balazs/LoRTIA.Our in-house scripts used to generate the descriptive statistics of reads and transcripts, to analyze promoters and to detect transcript isoforms are available on GitHub: https://github.com/moldovannorbert/seqtools. The sequencing datasets generated in this study are available at the European Nucleotide Archive SRA database under the accession PRJEB24943 & PRJEB27818 (https://www.ebi.ac.uk/ena/browser/view/PRJEB24943, https://www.ebi.ac.uk/ena/browser/view/PRJEB27818).

## References

[1] Blissard GW, Rohrmann GF (1990). Baculovirus diversity and molecular biology. Annu. Rev. Entomol..

[2] Tian JH (2021). SARS-CoV-2 spike glycoprotein vaccine candidate NVX-CoV2373 immunogenicity in baboons and protection in mice. Nat. Commun..

[3] Volkman LE, Summers MD, Hsieh CH (1976). Occluded and nonoccluded nuclear polyhedrosis virus grown in *Trichoplusia ni*: Comparative neutralization comparative infectivity, and in vitro growth studies. J. Virol..

[4] Ayres MD, Howard SC, Kuzio J, Lopez-Ferber M, Possee RD (1994). The complete DNA sequence of *Autographa californica* nuclear polyhedrosis virus. Virology.

[5] Rohrmann, G. Baculovirus molecular biology baculovirus molecular biology baculovirus molecular biology. *Baculovirus Mol. Biol.* 1–2 (2008).

[6] Guarino LA, Summers MD (1986). Functional mapping of a trans-activating gene required for expression of a baculovirus delayed-early gene. J. Virol..

[7] Kool M, Ahrens CH, Goldbach RW, Rohrmann GF, Vlak JM (1994). Identification of genes involved in DNA replication of the *Autographa californica* baculovirus. Proc. Natl. Acad. Sci. U. S. A..

[8] Kogan PH, Chen X, Blissard GW (1995). Overlapping TATA-dependent and TATA-independent early promoter activities in the baculovirus gp64 envelope fusion protein gene. J. Virol..

[9] Lu A, Carstens EB (1993). Immediate-early baculovirus genes transactivate the p143 gene promoter of *Autographa californica* nuclear polyhedrosis virus. Virology.

[10] Kovacs GR, Guarino LA, Graham BL, Summers MD (1991). Identification of spliced baculovirus RNAs expressed late in infection. Virology.

[11] Garrity DB, Chang M-J, Blissard GW (1997). Late promoter selection in the Baculovirusgp64 envelope fusion ProteinGene. Virology.

[12] Chen Y-R (2013). The transcriptome of the baculovirus *Autographa californica* multiple nucleopolyhedrovirus in *Trichoplusia ni* cells. J. Virol..

[13] Moldován N (2018). Third-generation sequencing reveals extensive polycistronism and transcriptional overlapping in a baculovirus. Sci. Rep..

[14] Ooi BG, Rankin C, Miller LK (1989). Downstream sequences augment transcription from the essential initiation site of a baculovirus polyhedrin gene. J. Mol. Biol..

[15] McLachlin JR, Miller LK (1994). Identification and characterization of vlf-1, a baculovirus gene involved in very late gene expression. J. Virol..

[16] Li Y, Guarino LA (2008). Roles of LEF-4 and PTP/BVP RNA triphosphatases in processing of baculovirus late mRNAs. J. Virol..

[17] Wu X, Guarino LA (2003). *Autographa californica* nucleopolyhedrovirus orf69 encodes an RNA cap (nucleoside-2′-O)-methyltransferase. J. Virol..

[18] Jin J, Guarino LA (2000). 3′-end formation of baculovirus late RNAs. J. Virol..

[19] Dubin DT, Taylor RH (1975). The methylation state of poly A-containing messenger RNA from cultured hamster cells. Nucleic Acids Res..

[20] Bataglia L, Simões ZLP, Nunes FMF (2021). Active genic machinery for epigenetic RNA modifications in bees. Insect Mol. Biol..

[21] Dubin DT, Stollar V (1975). Methylation of Sindbis virus “26S” messenger RNA. Biochem. Biophys. Res. Commun..

[22] Lavi S, Shatkin AJ (1975). Methylated simian virus 40-specific RNA from nuclei and cytoplasm of infected BSC-1 cells. Proc. Natl. Acad. Sci. U. S. A..

[23] Helm M (2006). Post-transcriptional nucleotide modification and alternative folding of RNA. Nucleic Acids Res..

[24] Kadaba S (2004). Nuclear surveillance and degradation of hypomodified initiator tRNAMet in *S. cerevisiae*. Genes Dev..

[25] Strobel MC, Abelson J (1986). Effect of intron mutations on processing and function of *Saccharomyces cerevisiae* SUP53 tRNA in vitro and in vivo. Mol. Cell. Biol..

[26] Vicens Q, Westhof E (2001). Crystal structure of paromomycin docked into the eubacterial ribosomal decoding A site. Structure.

[27] Karikó K, Buckstein M, Ni H, Weissman D (2005). Suppression of RNA recognition by toll-like receptors: The impact of nucleoside modification and the evolutionary origin of RNA. Immunity.

[28] Hussain S (2013). NSun2-mediated cytosine-5 methylation of vault noncoding RNA determines its processing into regulatory small RNAs. Cell Rep..

[29] Walkley CR, Li JB (2017). Rewriting the transcriptome: Adenosine-to-inosine RNA editing by ADARs. Genome Biol..

[30] Mannion NM (2014). The RNA-editing enzyme ADAR1 controls innate immune responses to RNA. Cell Rep..

[31] Zahn RC, Schelp I, Utermöhlen O, von Laer D (2007). A-to-G hypermutation in the genome of lymphocytic choriomeningitis virus. J. Virol..

[32] Wong SK, Lazinski DW (2002). Replicating hepatitis delta virus RNA is edited in the nucleus by the small form of ADAR1. Proc. Natl. Acad. Sci..

[33] Kukurba KR, Montgomery SB (2015). RNA sequencing and analysis. Cold Spring Harb. Protoc..

[34] Oláh P (2015). Characterization of pseudorabies virus transcriptome by Illumina sequencing. BMC Microbiol..

[35] Liu L (2012). Comparison of next-generation sequencing systems. J. Biomed. Biotechnol..

[36] Heather JM, Chain B (2016). The sequence of sequencers: The history of sequencing DNA. Genomics.

[37] Clarke J (2009). Continuous base identification for single-molecule nanopore DNA sequencing. Nat. Nanotechnol..

[38] Garalde DR (2018). Highly parallel direct RNA sequencing on an array of nanopores. Nat. Methods.

[39] Manrao EA (2012). Reading DNA at single-nucleotide resolution with a mutant MspA nanopore and phi29 DNA polymerase. Nat. Biotechnol..

[40] Laver T (2015). Assessing the performance of the Oxford Nanopore Technologies MinION. Biomol. Detect. Quantif..

[41] Moldován N (2017). Multi-platform sequencing approach reveals a novel transcriptome profile in pseudorabies virus. Front. Microbiol..

[42] Balázs Z, Tombácz D, Szűcs A, Snyder M, Boldogkői Z (2017). Long-read sequencing of the human cytomegalovirus transcriptome with the Pacific Biosciences RSII platform. Sci. Data.

[43] Smith AM, Jain M, Mulroney L, Garalde DR, Akeson M (2017). Reading canonical and modified nucleotides in 16S ribosomal RNA using nanopore direct RNA sequencing. bioRxiv.

[44] Stoiber MH (2017). De novo identification of DNA modifications enabled by genome-guided nanopore signal processing. bioRxiv.

[45] Smith I (2007). Misleading messengers? Interpreting baculovirus transcriptional array profiles. J. Virol..

[46] Jiang SS (2006). Temporal transcription program of recombinant *Autographa californica* multiple nucleopolyhedrosis virus. J. Virol..

[47] Microsoft Corporation. Microsoft PowerPoint. https://www.microsoft.com/hu-hu/microsoft-365/powerpoint (2021).

[48] Microsoft Corporation. Microsoft Excel. https://office.microsoft.com/excel (2021).

[49] Crooks GE, Hon G, Chandonia JM, Brenner SE (2004). WebLogo: A sequence logo generator. Genome Res..

[50] Kearse M (2012). Geneious basic: An integrated and extendable desktop software platform for the organization and analysis of sequence data. Bioinformatics.

[51] Calvo SE, Pagliarini DJ, Mootha VK (2009). Upstream open reading frames cause widespread reduction of protein expression and are polymorphic among humans. Proc. Natl. Acad. Sci. U. S. A..

[52] Kronstad LM, Brulois KF, Jung JU, Glaunsinger BA (2013). Dual short upstream open reading frames control translation of a herpesviral polycistronic mRNA. PLoS Pathog..

[53] Wickham H (2016). ggplot2: Elegant graphics for data analysis.

[54] Matthews MM (2016). Structures of human ADAR2 bound to dsRNA reveal base-flipping mechanism and basis for site selectivity. Nat. Struct. Mol. Biol..

[55] Thorvaldsdóttir H, Robinson JT, Mesirov JP (2013). Integrative Genomics Viewer (IGV): High-performance genomics data visualization and exploration. Brief. Bioinform..

[56] Tombácz D (2017). Long-read isoform sequencing reveals a hidden complexity of the transcriptional landscape of herpes simplex virus type 1. Front. Microbiol..

[57] Depledge DP (2019). Direct RNA sequencing on nanopore arrays redefines the transcriptional complexity of a viral pathogen. Nat. Commun..

[58] Torma G (2021). Combined short and long-read sequencing reveals a complex transcriptomic architecture of African Swine Fever Virus. Viruses.

[59] Shine J, Dalgarno L (1975). Determinant of cistron specificity in bacterial ribosomes. Nature.

[60] Boldogkői Z, Moldován N, Balázs Z, Snyder M, Tombácz D (2019). Long-read sequencing—A powerful tool in viral transcriptome research. Trends Microbiol..

[61] Boldogköi Z (2012). Transcriptional interference networks coordinate the expression of functionally related genes clustered in the same genomic loci. Front. Genet..

[62] Vilela C, McCarthy JEG (2003). Regulation of fungal gene expression via short open reading frames in the mRNA 5′untranslated region. Mol. Microbiol..

[63] Matoulkova E, Michalova E, Vojtesek B, Hrstka R (2012). The role of the 3′ untranslated region in post-transcriptional regulation of protein expression in mammalian cells. RNA Biol..

[64] Pearson M, Bjornson R, Pearson G, Rohrmann G (1992). The Autographa californica baculovirus genome: Evidence for multiple replication origins. Science (80-)..

[65] van Oers M, Vlak J (2007). Baculovirus genomics. Curr. Drug Targets.

[66] Carstens EB, Wu Y (2007). No single homologous repeat region is essential for DNA replication of the baculovirus *Autographa californica* multiple nucleopolyhedrovirus. J. Gen. Virol..

[67] Boldogkői Z, Balázs Z, Moldován N, Prazsák I, Tombácz D (2019). Novel classes of replication-associated transcripts discovered in viruses. RNA Biol..

[68] Tombácz D (2016). Full-length isoform sequencing reveals novel transcripts and substantial transcriptional overlaps in a herpesvirus. PLoS One.

[69] Torma G (2021). An integrated sequencing approach for updating the pseudorabies virus transcriptome. Pathogens.

[70] Yang X (2017). 5-methylcytosine promotes mRNA export-NSUN2 as the methyltransferase and ALYREF as an m 5 C reader. Cell Res..

[71] Shi M (2017). ALYREF mainly binds to the 5′ and the 3′ regions of the mRNA in vivo. Nucleic Acids Res..

[72] Nishikura K (2006). Editor meets silencer: Crosstalk between RNA editing and RNA interference. Nat. Rev. Mol. Cell Biol..

[73] Boldogkői Z, Moldován N, Szűcs A, Tombácz D (2018). Data descriptor: Transcriptome-wide analysis of a baculovirus using nanopore sequencing. Sci. Data.

[74] Bayega, A. *et al.* Chapter 6. **1783**, (2018).

[75] Li H (2018). Minimap2: Pairwise alignment for nucleotide sequences. Bioinformatics.

