# Supplementary material for: Dual isoform sequencing reveals complex transcriptomic and epitranscriptomic landscapes of a prototype baculovirus
Source: Sci Rep. 2022 Jan 25;12:1291. doi: 10.1038/s41598-022-05457-8 (PMC8789824; doi:10.1038/s41598-022-05457-8)
Supplement: Supplementary file 7 — Supplementary Figures. [file 41598_2022_5457_MOESM7_ESM.docx]

**Dual Isoform Sequencing Reveals Complex Transcriptomic and Epitranscriptomic Landscapes of a Prototype Baculovirus**

Gábor Torma, Dóra Tombácz, Norbert Moldován, Ádám Fülöp, István Prazsák, Zsolt Csabai, Michael Snyder, and Zsolt Boldogkői


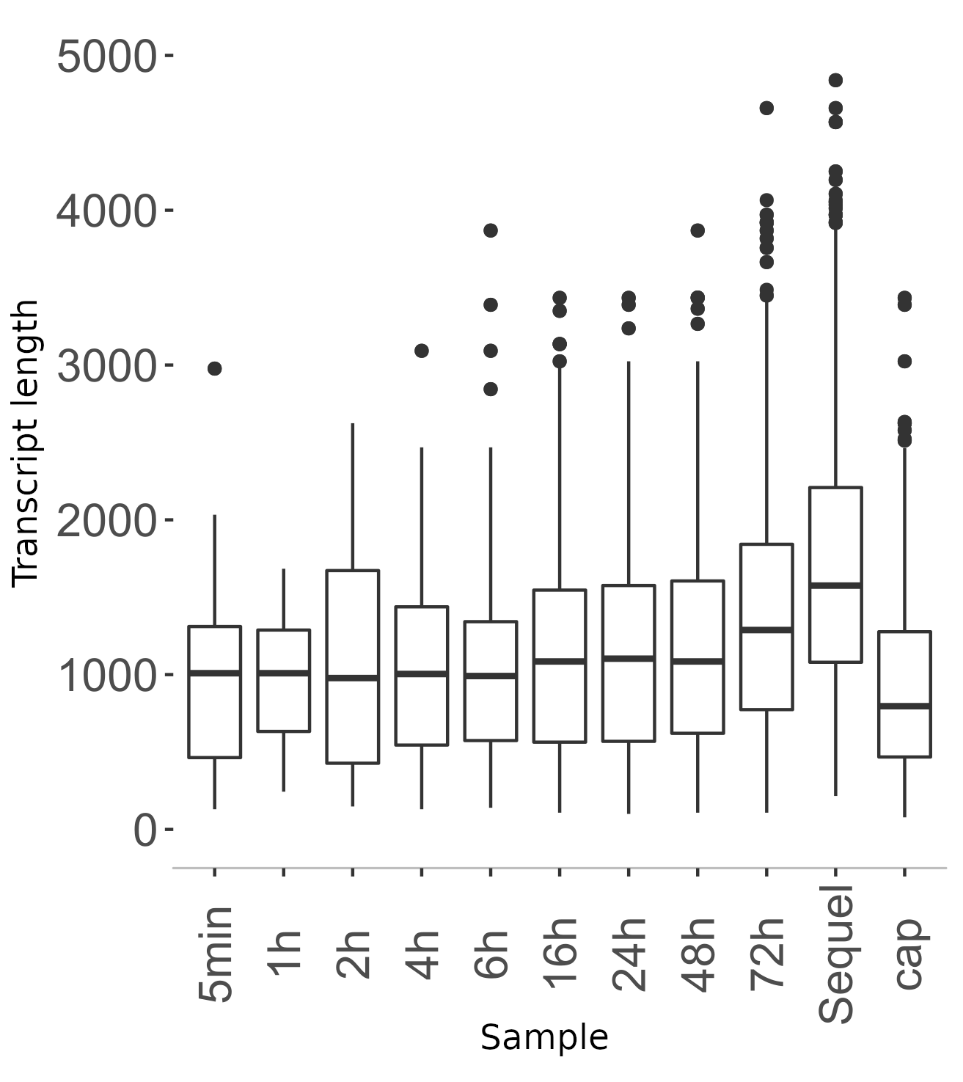


**Supplementary figure 1.** Boxplot distribution of transcript reads.


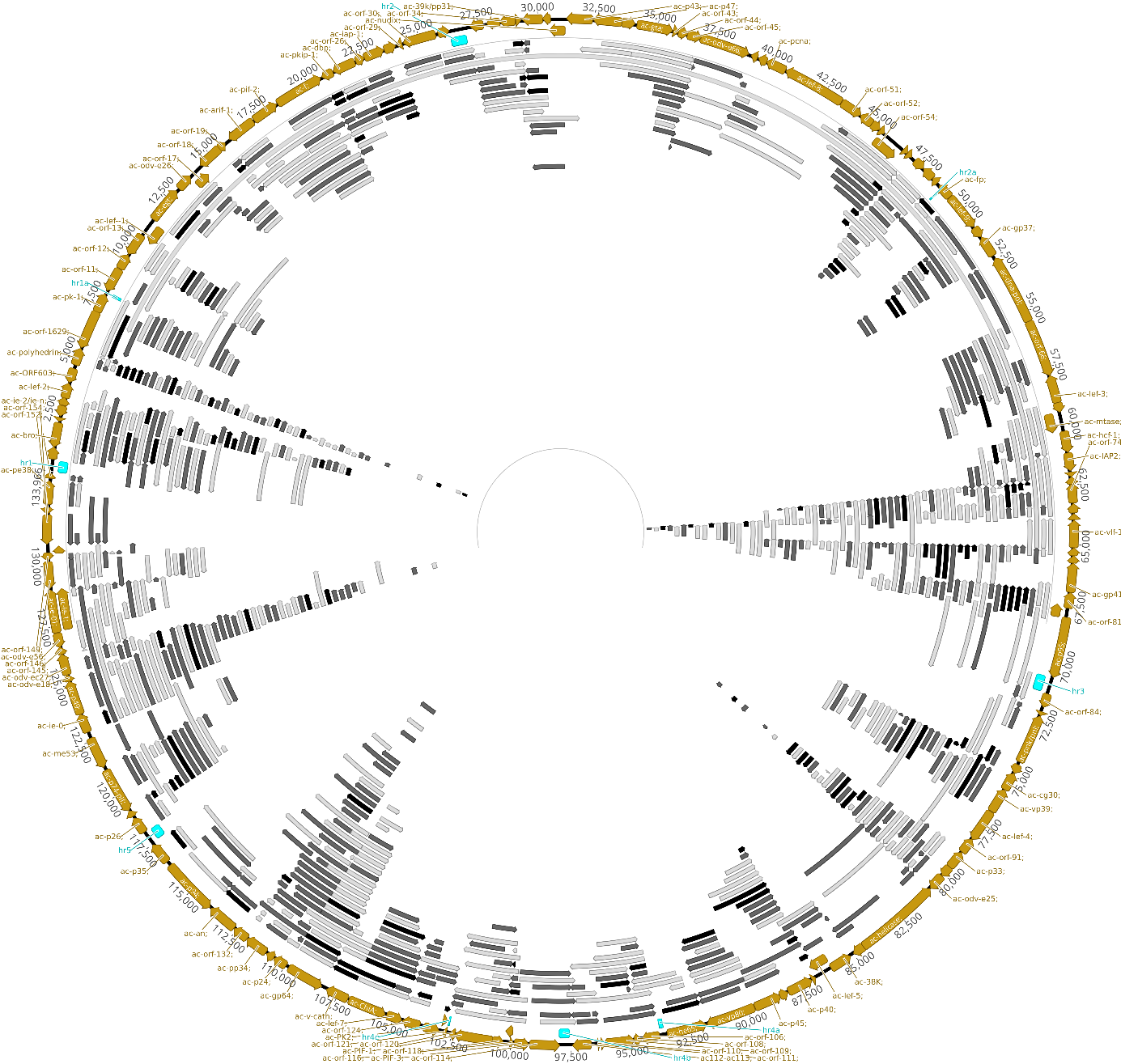


**Supplementary figure 2.** Semi-quantitative abundance of AcMNPV transcripts. Color code: light grey: low-abundance transcripts (1-5 reads); dark grey: medium-abundance transcripts (6-10 reads); high-abundance transcripts (11 – 10,000 reads). Single-read transcripts were identified on the basis of multiple TSSs and/or TESs by the LoRTIA suit.


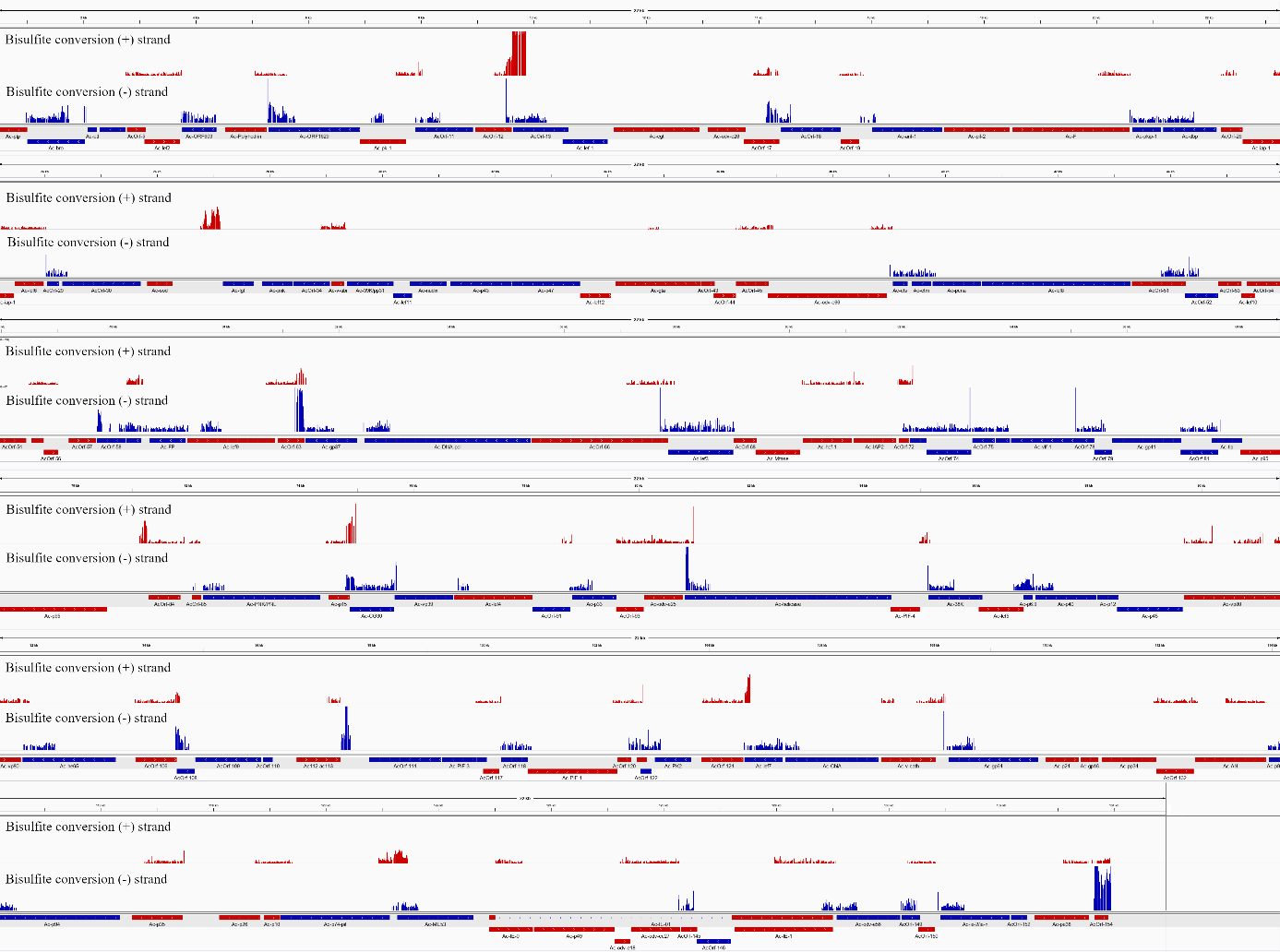


**Supplementary figure 3.** Distribution of 5-methylcytosines along the AcMNPV genome. Red peaks show methylation on the positive DNA strand and blue peaks on the negative strand.
